# Based on digital intelligence: teaching innovation and practice of veterinary internal medicine in China’s southwest frontier

**DOI:** 10.3389/fvets.2025.1651179

**Published:** 2025-09-24

**Authors:** Meiquan Li, Xiao Wang, Yanli Du, Hongyan Zhang, Bo Liao

**Affiliations:** College of Agriculture and Life Sciences, Kunming University, Kunming, China

**Keywords:** veterinary internal medicine, digital intelligence, integration of online and offline modalities, teaching innovation, China’s southwest frontier

## Abstract

Veterinary internal medicine is a core discipline in animal medicine, crucial for advancing the livestock industry. Nevertheless, conventional teaching methods often suffer from fragmented content, inadequate clinical exposure, and a disconnect from industry requirements. In response, reforms guided by the principle of “integrating knowledge with practice and harmonizing learning with application” are increasingly adopting digital and intelligent technologies. Key innovations include the use of AI-based knowledge graphs to reorganize curricula into a three-phase virtual-real closed-loop system, enabling blended online-offline instruction. This structure supports the integration of theoretical learning, virtual simulation, and clinical application through case analyses, practical exercises, and collaborative discussions. Additionally, virtual simulation platforms and academic-industry partnerships provide holistic training via intelligent case studies and realistic scenario simulations, enhancing clinical reasoning and operational skills. A multidimensional dynamic assessment system has also been introduced, incorporating process-oriented and multi-source evaluations. This system blends online and offline components, integrates corporate feedback, embeds public health and ideological education, and employs blockchain-based micro-credentials to certify competencies. These developments contribute to improved diagnostic decision-making and professional aptitude, while also facilitating the digital transformation of the livestock sector by cultivating skilled, innovative practitioners.

## Introduction

Veterinary Internal Medicine is a fundamental discipline within veterinary education, playing a critical role in bridging basic medical knowledge and clinical practice. As a core course for third-year veterinary students, it focuses on the pathogenesis, diagnosis, and treatment of internal diseases in animals, aiming to develop well-rounded professionals with solid theoretical grounding, clinical reasoning skills, and innovative capabilities. Globally, veterinary education is increasingly emphasizing competency-based training and the integration of digital technologies to enhance clinical preparedness and regional adaptability.

In China, particularly in the southwestern border regions, the veterinary profession faces unique challenges due to distinctive geographical and climatic conditions, such as high-altitude environments and cross-border disease transmission risks. These regional characteristics demand tailored educational approaches that align with local livestock industry development and disease control needs.

**Figure 1:**
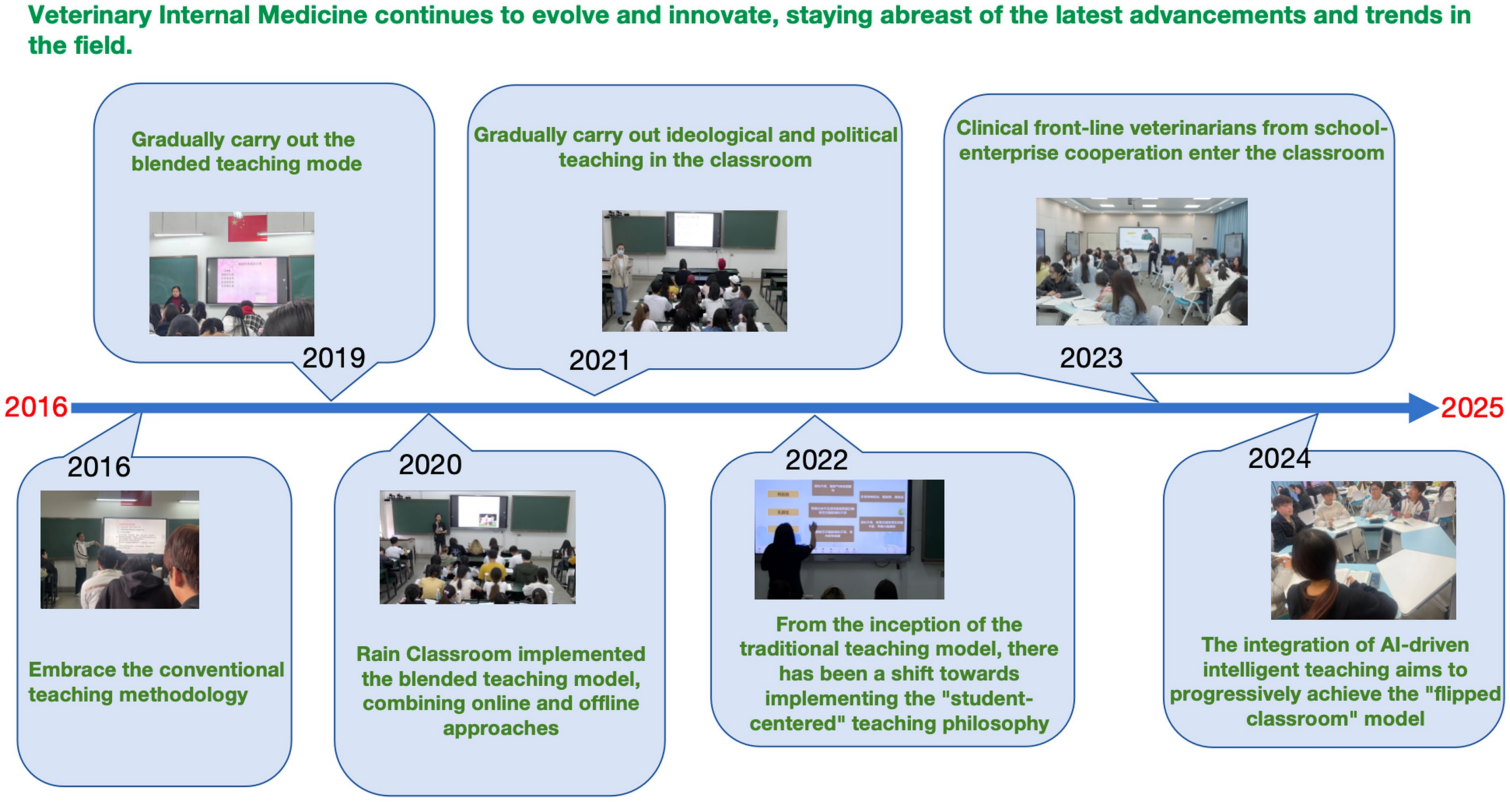
The development process of veterinary internal medicine.

Take Kunming University as an example, the course development can be divided into three phases. In the initial phase, a traditional teaching model was adopted, consisting of 54 theoretical and 10 experimental hours, with an emphasis on consolidating teaching resources. Beginning in 2019, the course entered a transformation phase: total hours were reduced to 42 theoretical and 6 experimental hours; the curriculum was restructured to incorporate national-level online open courses, ideological-political cases, and regional monographs specific to the southwestern border areas. A blended learning model was gradually introduced. By 2022, the course entered a formative phase centered on student-oriented learning, establishing a tiered teaching framework of “online preview – offline interaction – post-class consolidation.” Further enhancements included deepened school-enterprise collaboration, integration of artificial intelligence (AI), and flipped classroom practices. From 2023 to 2024, the course gained recognition as a university-level first-class course, a provincial-level blended first-class course, and a university-level ideological-political demonstration course. This evolution reflects a shift from knowledge dissemination to competence cultivation, achieving deep integration of regional characteristics and technological innovation (Figure 1).

Guided by veterinary educational principles and student learning profiles, the teaching team has actively incorporated local industry developments and employer feedback—especially regarding professional competency requirements—to continuously refine an innovative teaching model that integrates AI with practical application in veterinary internal medicine. This initiative aims to strengthen graduates’ employability, drive updates in teaching content and resources, foster pedagogical innovation, and diversify evaluation methods, ultimately forming a closed-loop continuous improvement mechanism for sustainable curriculum development.

## Challenges in the instruction of veterinary internal medicine

### Analysis of course learning

In veterinary medical education, the pathophysiological parallels between animal and human diseases provide a valuable framework for instructional design (1). These similarities facilitate the comprehension of complex veterinary concepts through analogical reasoning, interdisciplinary integration, and clinical empathy (2). Numerous conditions including viral infections, diabetes mellitus, cardiovascular diseases, and renal pathologies exhibit mechanistic commonalities across species, such as vaccine-induced immunity, insulin resistance, atherosclerotic progression, and glomerular dysfunction. By building upon students’ prior knowledge in biology and human medicine, for instance, the circulatory system as taught in secondary education or health topics encountered in everyday contexts, instructors can effectively elucidate veterinary pathologies through comparative analogy. This approach promotes deeper conceptual understanding and supports knowledge transfer between human and animal health domains.

The course in Veterinary Internal Medicine is typically delivered in the first semester of the third year within the veterinary curriculum. By this stage, students have acquired foundational scientific knowledge and developed preliminary competencies in self-directed learning, which together provide a suitable basis for engaging with more integrated and clinically oriented content (3).

However, despite the inherent educational advantages offered by comparative medicine, several pedagogical challenges have been observed in the teaching of Veterinary Internal Medicine (4). These include variability in students’ foundational knowledge, limited clinical reasoning skills, and suboptimal engagement with self-directed learning activities (5). These issues often hinder the achievement of desired learning outcomes and alignment with course objectives.

To better understand the learning profile of incoming students, a pre-course survey was administered assessing motivation, prior knowledge, and understanding of comparative disease mechanisms in humans and animals. The survey aimed to identify learning needs, expected outcomes, baseline knowledge levels, and current learning behaviors. Data collected informed the refinement of course objectives and supported the development of targeted instructional strategies for Veterinary Internal Medicine (6).

Through longitudinal teaching practice and evidence-based reflection, a multi-dimensional analytical framework was developed. This system integrates dynamic learning analytics, teaching outcome correlations, reflective textual analysis, and cross-disciplinary collaboration to transition from subjective impression to data-informed identification of instructional challenges. This approach has enabled precise diagnosis of common barriers to student learning (7, 8). The following specific learning difficulties associated with Veterinary Internal Medicine have been identified:

## Pain point 1: fragmentation of theoretical knowledge and weak integrative capacity

### Cognitive overload

Veterinary internal medicine involves complex multi-system diseases and interdisciplinary pathogenic mechanisms. Conventional linear instructional approaches often lead to fragmented knowledge acquisition, which impedes students’ ability to construct a coherent and systematic knowledge framework (9). Furthermore, an overreliance on rote memorization of disease classifications, such as categorizing “porcine pancreatitis solely under digestive system diseases,” fails to capture its associations with metabolic dysregulation and immune activation (10).

### Accumulation of inert knowledge

Students can often recite factual information, such as “glucocorticoids are used for anti-inflammatory purposes,” yet demonstrate limited capacity to analyze underlying pharmacological targets. For instance, inhibition of the NF-κB pathway, dose–response characteristics, or interspecies variations in drug efficacy (11). Similarly, instruction regarding disease mechanisms frequently remains descriptive (e.g., noting elevated blood ammonia in hepatic encephalopathy) without integrating deeper causal chains spanning etiology, molecular pathways, clinical manifestations, and therapeutic interventions. This superficial understanding constrains learners’ ability to transfer and apply knowledge in clinical contexts (12).

Students frequently demonstrate the ability to recall factual information, such as “glucocorticoids exert anti-inflammatory effects,” yet exhibit limited capacity for mechanistic analysis. Specifically, they struggle to interrogate underlying pharmacological targets—for instance, the inhibition of the NF-κB signaling pathway (13), dose–response relationships, or interspecies variations in drug metabolism and efficacy. Moreover, instruction pertaining to disease mechanisms often remains superficial, emphasizing descriptive phenomena (e.g., elevated blood ammonia levels in hepatic encephalopathy) without articulating a coherent continuum spanning etiology, molecular pathogenesis, clinical manifestations, and therapeutic targeting. This conceptual discontinuity significantly constrains knowledge transfer and practical application in clinical settings.

## Pain point 2: deficiencies in clinical reasoning and situational transfer ability

### Limited differential diagnosis capability

When presented with cases exhibiting multiple clinical signs—such as fever, cough, and anorexia—students often struggle to construct and prioritize a structured “symptom tree.” For instance, even after initially ruling out zoonotic diseases, learners tend to enumerate all possible disorders without adequately assessing clinical relevance or epidemiological probability. Moreover, in the context of multifactorial diseases such as bovine ketosis (involving negative energy balance, endocrine dysregulation, and management factors), students frequently attribute causation to a single element—e.g., “glucose deficiency”—while overlooking complex interactions among environmental influences, host status, and pathogenic mechanisms (14).

### Homogenization of treatment strategies

A significant blind spot exists in the individualization of diagnosis and therapy. Students often apply standardized treatment protocols mechanistically; for example, calculating antibiotic dosages based solely on body weight without incorporating critical variables such as breed, physiological state, or metabolic differences (15, 16). Furthermore, there is a conspicuous lack of dynamic clinical thinking, resulting in failure to adapt treatment plans in response to therapeutic feedback or evolving clinical conditions.

## Pain point 3: inadequate learning motivation and lack of professional identity

### Underutilization of achievement motivation

The predominant reliance on standardized written examinations, which prioritize memorization of factual knowledge, fails to effectively evaluate higher-order clinical competencies such as case analysis and clinical decision-making. This assessment approach encourages a strategic orientation toward minimal proficiency rather than promoting deep, conceptual learning. Furthermore, insufficient engagement with authentic clinical scenarios within the veterinary internal medicine curriculum reduces students’ perception of professional relevance and diminishes their sense of vocational mission (15).

## Innovative educational concepts and strategic interventions

To address the pedagogical challenges identified above, an instructional framework centered on “student development, professional competency enhancement, and regional service integration” has been proposed. By systematically integrating knowledge structures, clinical workflows, value cultivation, and multi-stakeholder collaboration, a holistic teaching ecosystem enabled by AI augmentation (17), virtual-real simulation, and cross-sectoral cooperation has been constructed. Furthermore, accounting for regional contextual factors such as those present in Yunnan the teaching of veterinary internal medicine has been strategically aligned with three core challenges: “knowledge fragmentation, underdeveloped clinical reasoning chains, and insufficient motivation activation” (18, 19). Accordingly, a set of evidence-based teaching intervention strategies has been formulated.

## AI-enhanced learning

AI is leveraged to intelligently reconfigure the pedagogy of disease mechanisms, optimizing knowledge delivery and enabling personalized learning trajectories and precision skill development (20–22). The field of veterinary medicine has limited and fragmented data and variable data quality, which limits the development and validation of AI models (23). But over-reliance on AI may lead to degradation of veterinary skills, for example in diagnosis and treatment, if over-reliance on AI may lead to a decline in clinical skills (24).

## Virtual-real integration

A blended practical teaching system combining virtual simulations and real clinical scenarios is established to mitigate limitations in clinical training resources. This approach enhances the acquisition of higher-order clinical competencies through synergistic virtual-physical experiential learning (25).

## Cross-sector collaborative education

An integrated educational ecosystem is constructed, facilitating collaboration among academic institutions, veterinary hospitals, and industry partners. This model breaks down resource barriers, capitalizes on complementary expertise, and fosters the development of interdisciplinary professionals capable of addressing complex industry challenges (26).

## Innovative methodologies and advanced instructional approaches in veterinary medicine

The teaching content was systematically restructured according to a “three-tier closed-loop, virtual-reality symbiotic” instructional model (27). To address Problem 1 (Fragmentation of Theoretical Knowledge and Weak Integration Skills), research-oriented learning strategies were implemented through the following measures:

1. Modular reorganization and knowledge granulation

The “digestive system diseases” module was reorganized into three thematic sub-modules: ruminant forestomach disorders, monogastric animal gastroenteritis, and nutritional metabolic diseases. A total of 20 fine-grained knowledge units were defined, including “pathophysiological mechanisms of ruminal acidosis” and “dosage calculation for propylene glycol therapy,” forming a structured knowledge network that facilitates cross-modular retrieval and comparative learning (28). For example, in a case study on parasitic infections in yaks at high altitude, an AI-enhanced case modeling system automatically integrated environmental parameters, such as regional altitude and pasture selenium content, to generate a dynamic clinical scenario incorporating multidimensional information: life cycle of *Fasciola hepatica*, cost-effectiveness of traditional anthelmintic agents, and ecological impact on grasslands. Students were tasked with synthesizing these knowledge chains to formulate diagnosis and treatment plans (29, 30).

1. Implementation of the “three-tier closed-loop” instructional approach

A structured teaching methodology was implemented to enhance knowledge integration and clinical reasoning capabilities. Before class, students received targeted pre-learning guidance through instructional containing embedded knowledge graph nodes, delivered via a digital learning platform. An AI-driven question-answering system analyzed online learning behaviors and assigned personalized preview tasks to promote self-directed learning (31, 32).

During class, a blended virtual-reality environment was established using dynamic knowledge maps to create immersive learning experiences (27). Instructors emphasized core knowledge points while students collaboratively constructed and refined concept maps using mind-mapping software (e.g., Xmind). Interlinkages among anatomical structures, metabolic pathways, and region-specific livestock data were highlighted (33). Real-time feedback on logical reasoning errors was provided through an interactive digital teaching system, which also generated visualizable performance metrics.

To simulate real-world clinical scenarios, students were assigned role-based tasks—such as veterinarians, livestock owners, and pharmacists—and worked in groups to complete timed exercises involving etiological, treatment design, and economic evaluation through a card-based task progression system (34, 35). Performance was evaluated based on logical coherence and interrole collaboration efficiency, with an incentive mechanism (“Veterinary Star” points) used to reward comprehensive performance. Furthermore, thematic debate sessions were organized with remote evaluation provided by industry experts via teleconferencing platforms (36–38).

Post-class knowledge transfer was facilitated through innovation-oriented projects, such as the design of compound ivermectin formulations incorporating traditional herbal medicine. Students utilized scientific databases to optimize component ratios and evaluate efficacy. An AI multimodal assessment system generated multidimensional radar maps evaluating performance across scientific rigor, practical feasibility, and ecological sustainability, with results integrated into the overall course evaluation (38, 39).

This integrated instructional strategy aims to bridge foundational, clinical, and frontier knowledge domains, fostering veterinary professionals with robust integrative abilities, practical adaptability, and innovative competencies (40, 41).

To address Problem 2 (Insufficient Clinical Reasoning and Situational Transferability), a comprehensive clinical training system was established through a tripartite collaboration involving academic institutions, animal hospitals, and industry partners (42). This partnership established a closed-loop mechanism integrating “resource sharing, practical training, and competency certification.”

A series of typical clinical scenarios—such as bovine ketosis cases—were developed using virtual simulation platforms. Students were tasked with comparing real-time diagnostic and treatment data collected from production settings throughout procedures such as blood ketone testing and pregnancy-safe medication selection. AI triggered micro-lectures delivered by practicing veterinarians, which addressed common clinical errors, for example, complications arising from excessive propylene glycol administration (43).

Animal hospitals provided anonymized case databases and live-streamed surgical procedures. Through a virtual consultation system, students simulated client interactions, with AI algorithms evaluating the completeness and logical coherence of their diagnostic inquiry. Simultaneously, live broadcasts of emergency interventions, such as correction of gastric torsion, were accompanied by picture-in-picture commentary from senior clinicians explaining critical decision points. Interactive bullet-screen technology allowed students to vote on treatment choices, with incorrect selections triggering immediate AI-generated feedback (44).

Industry partners contributed ranch IoT data streams and supported a “one-trainee-one-plan” approach, enabling personalized training schedules. Students undertook monthly 24-h on-site shifts at production facilities, where they integrated portable ketone meter readings and perinatal metabolic profiles to develop individualized intervention strategies. A dynamic reinforcement module provided targeted training in identified areas of weakness and exposed learners to exemplary clinical practices based on performance analytics (45).

Upon joint evaluation by all three stakeholders, participants were awarded digital skill badges certifying their clinical competencies. Those certified received priority access to internship opportunities at partner institutions. Pilot implementation data indicated that participants’ clinical decision-making accuracy improved from 58 to 86% (46). These outcomes demonstrate the efficacy of a pedagogy that transforms traditional classrooms into clinical training environments and integrates learning with practical application.

## The entire process is comprehensively engaged in constructing a four-dimensional dynamic evaluation system

To address Problem 2 (Insufficient Clinical Reasoning and Situational Transferability), a process-oriented, multi-competency certification model was implemented through a “4-3-3” evaluation framework. This system integrates dynamic assessment across theoretical, practical, and innovative dimensions to holistically evaluate student performance.

1. Dynamic multi-dimensional evaluation structure

The evaluation system consists of three weighted components: A written theoretical examination (40%) assessing knowledge of disease mechanisms and diagnostic-therapeutic criteria; An in-class practical evaluation (30%) involving role-playing tasks based on a Clinical Decision Tree system; An online assessment (30%) comprising virtual case analysis (10%), real-world diagnostic tasks (10%), and innovation proposals (10%) (47). The virtual case module utilizes a borderline case database wherein students are required to diagnose and treat conditions such as rumen acidosis in yaks within a 30-min timeframe with AI assistance. The system tracks operational pathways in real time; deviations from clinical logic—such as omitting anatomical consultation of the forestomach 3D model and proceeding directly to ethylene oxide therapy—trigger automated feedback and score deductions. Concurrently, context-aware micro-lessons (e.g., on traditional herbal regulation of rumen microbiota) are delivered to address knowledge gaps. Real diagnostic tasks are connected to IoT platforms from partnering biotech companies, allowing students to analyze real-time blood ketone data from dairy cattle and develop individualized treatment plans under realistic cost constraints. Enterprise mentors evaluate submissions based on scientific validity, economic feasibility, and operational practicality (48).

1. Innovation and professional certification

For the innovation segment, students undertake projects such as “Comparative Efficacy of Tibetan Bitter Herbs and Ivermectin in Deworming.” Tasks include database mining (e.g., NCBI), component analysis (LC–MS), ecological impact assessment, egg reduction rate testing, and grassland microbial diversity evaluation. Final reports incorporate cost–benefit analysis, therapeutic thresholds, and ecological metrics, and are evaluated through triple-blind review by academic and industry mentors. A “Star Veterinarian” badge system certifies professional competencies. For example, students who design science communication materials for community zoonosis prevention during a 24-h documentary task can earn a “Zoonotic Disease Control Pioneer” badge. Accumulation of three badges qualifies students for direct internship placements with partner enterprises. Pilot assessment data show that the clinical logic error rate among the 2023 cohort decreased by 41, and 27% of Tibetan medicine-based comparative studies were adopted for field trials by partner ranches. These results affirm the efficacy of the evaluation system in enhancing practical innovation capabilities and supporting translational learning outcomes (49).

## The “Three-Dimensional Linkage” approach enhances the efficacy of ideological and political education

The “Three-Dimensional Linkage” approach enhances the effectiveness of ideological and political education within the veterinary curriculum by systematically integrating localized, contextual, and values-driven content into professional training. Recognizing that a significant proportion of students originate from within the region, a dedicated case repository aligned with local demographics, events, and material culture has been developed. This repository supports instruction by embedding regionally relevant examples into pedagogical practices, thereby strengthening students’ identification with course content and increasing intrinsic motivation.

Ideological and political elements are incorporated through a multi-level classroom structure:

In-class teaching employs case analyses, structured debates, student-led demonstrations, and role-playing exercises. For example, real-world cases such as rabies control in border areas are used to illustrate veterinarians’ social responsibilities within a “One Health” framework. Documentary footage showcasing frontline veterinary efforts during disease outbreaks helps cultivate a sense of professional mission and ethical accountability. Controversial topics, such as balancing low-cost, high-efficiency drug use against high-cost animal welfare considerations, are addressed through guided debates, encouraging students to reflect on economic, ethical, and ecological trade-offs (50).

Outstanding students demonstrate complete diagnostic processes, while peers assume roles such as veterinarians, pet owners, and laboratory technicians to simulate clinical interactions (51). This method promotes empathy, communication skills, and ethical reasoning, achieving integration across knowledge, skills, and values (52).

The secondary classroom, often situated in a campus animal hospital setting, provides simulated diagnostic and treatment tasks. Designed activities, such as a zoonotic disease prevention experiment, allow students to engage with complex professional scenarios, including managing conflicts with animal owners, thereby deepening their understanding of professional duties in a controlled yet realistic environment (53–55).

However, the described pedagogical approach, while innovative and comprehensive, exhibits potential drawbacks including over-reliance on technology infrastructure, which may exacerbate educational inequities in under-resourced settings. The complex multi-modal assessment system risks creating evaluative fragmentation, where standardized competency measurement becomes challenging across diverse digital and practical domains. Furthermore, the intensive resource requirements, including AI platforms, IoT infrastructure, and industry partnerships, render scalability difficult and potentially unsustainable for widespread implementation. The emphasis on technological engagement may inadvertently diminish fundamental clinical skill development through direct hands-on experience with animals.

## Conclusion

In summary, this educational reform successfully addresses key challenges in veterinary internal medicine instruction by leveraging digital and intelligent technologies. Through a restructured blended-learning framework, enhanced virtual-real simulation training, and a multidimensional evaluation system, students’ clinical reasoning, practical capabilities, and professional competence have been significantly improved. These advancements effectively bridge the gap between academic training and industry needs, contributing to the cultivation of talent capable of supporting the intelligent transformation of the livestock sector.
